# Assessment of goal-directed behavior and prospective memory in adult ADHD with an online 3D videogame simulating everyday tasks

**DOI:** 10.1038/s41598-023-36351-6

**Published:** 2023-06-08

**Authors:** Jussi Jylkkä, Liisa Ritakallio, Liya Merzon, Suvi Kangas, Matthias Kliegel, Sascha Zuber, Alexandra Hering, Matti Laine, Juha Salmi

**Affiliations:** 1 Department of Psychology, Åbo Akademi University, Turku, Finland; 2 Department of Neuroscience and Biomedical Engineering, Aalto University, Helsinki, Finland; 3 Department of Psychology and Logopedics, University of Helsinki, Helsinki, Finland; 4 Faculty of Psychology and Educational Sciences, University of Geneva, Geneva, Switzerland; 5 Centre for the Interdisciplinary Study of Gerontology and Vulnerability, University of Geneva, Geneva, Switzerland; 6 Swiss National Center of Competences in Research LIVES—Overcoming Vulnerability: Life Course Perspectives, Lausanne, Geneva, Switzerland; 7 School for Social and Behavioral Sciences, Department of Developmental Psychology, Tilburg University, Tilburg, The Netherlands; 8 Cognitive Aging Lab, Faculty of Psychology and Educational Sciences, University of Geneva, Geneva, Switzerland

**Keywords:** Psychology, Human behaviour

## Abstract

The diagnosis of ADHD is based on real-life attentional-executive deficits, but they are harder to detect in adults than in children and objective quantitative measures reflecting these everyday problems are lacking. We developed an online version of EPELI 3D videogame for naturalistic and scalable assessment of goal-directed action and prospective memory in adult ADHD. In EPELI, participants perform instructed everyday chores in a virtual apartment from memory. Our pre-registered hypothesis predicted weaker EPELI performances in adult ADHD compared to controls. The sample comprised 112 adults with ADHD and 255 neurotypical controls comparable in age (mean 31, SD = 8 years), gender distribution (71% females) and educational level. Using web-browser, the participants performed EPELI and other cognitive tasks, including Conner’s Continuous Performance Test (CPT). They also filled out questionnaires probing everyday executive performance and kept a 5-day diary of everyday prospective memory errors. Self-reported strategy use in the EPELI game was also examined. The ADHD participants’ self-ratings indicated clearly more everyday executive problems than in the controls. Differences in the EPELI game were mostly seen in the ADHD participants’ higher rates of task-irrelevant actions. Gender differences and a group × gender interaction was found in the number of correctly performed tasks, indicating poorer performance particularly in ADHD males. Discriminant validity of EPELI was similar to CPT. Strategy use strongly predicted EPELI performance in both groups. The results demonstrate the feasibility of EPELI for online assessment and highlight the role of impulsivity as a distinctive everyday life problem in adult ADHD.

## Introduction

Attention deficit hyperactivity disorder (ADHD) is diagnosed on the basis of a variety of attentional executive deficits hampering everyday life that span the three broad domains of inattention, hyperactivity, and impulsivity^1^. Even though ADHD symptoms typically manifest in real-world situations with multiple simultaneous attentional executive demands (e.g. situations where attending, ignoring, remembering and planning are needed), objective assessment methods typically focus on measuring a single rather narrow cognitive domain at a time (e.g. inhibition). This could be one reason why objective methods are generally considered to have a limited role in clinical characterization of ADHD^2,3^. It has been proposed that more ecologically valid paradigms simulating real-world situations where the symptoms are manifested are needed to quantify the challenges that individuals with ADHD may encounter in their everyday lives^4–7^.

Some of the symptoms, especially hyperactivity, are diminished during the course of development, making detection of ADHD more difficult in adults than in children^8^. Only about 15% of the ADHD cases detected at childhood still meet the criteria for a diagnosis at adulthood, but about 65% of the cases show signs of residual impairment^9,10^. Overall, the prevalence of adult ADHD is approximately 2.6%^11^. According to some sources, only about half of the neuropsychological studies report clear cognitive deficits in ADHD adults^12–14^. Besides heterogeneity in the symptoms and mismatch between real-life problems and isolated tasks, detection of adult ADHD is complicated by the high prevalence of various comorbid disorders such as mood disorders^15^, which also appear to be different between females and males^16,17^. Moreover, there is evidence that the core ADHD symptoms in males are more noticeable and easier to detect than in females^18^. Finally, the role of possible compensatory strategies to manage situations with high executive demands may also increase from childhood to adulthood^19,20^. All aforementioned factors make adult ADHD a challenging target for reliable objective measurements.

One cognitive construct that covers various aspects of everyday life attentional executive functions is prospective memory (PM)^21^. As almost any goal-directed behavior relates to ‘future actions’, the umbrella of PM has been noted to explain more than 50–70% of the memory failures in everyday life^22,23^. There are some studies examining the role of specific aspects of PM problems observed in ADHD, especially monitoring and doing things in time, and reacting to external memory cues^24–28^. As PM is needed basically in all sorts of everyday goal-directed behaviors, there are also various attentional executive functions such as multitasking or monitoring that support PM performance and can be quantified in a PM task.

Shallice and Burgess developed a framework for PM studies where the participants perform several everyday memory tasks in real surroundings (e.g. navigate in a shopping mall to do a set of parallel tasks, meet certain people at a certain time, buy some objects from a supermarket)^29^. Inspired by their Multiple Errands Test and its virtual reality (VR) variant^30^, we have recently developed the EPELI (Executive Performance of Everyday Living) task employing game technology and operationalized several metrics to capture challenges that individuals with ADHD have in their daily life^7^. With the original head-mounted display (HMD) version of EPELI developed for studying children, we demonstrated that ADHD in school-age children is associated with a lower number of correctly performed tasks, poorer task efficacy (the proportion of relevant actions out of all actions) and navigation efficacy (total score divided by distance covered in the apartment), higher activity level (indexed by Controller motion), and higher rates of all actions^7^.

In this preregistered study (see Study 1 in https://osf.io/3y5je/), we used a Web browser -based version of the new adult EPELI^31^ to study the sensitivity of this task in detecting cognitive deficits in ADHD adults in a large-enough sample that permits exploring the role of important background factors. In addition to EPELI, the participants conducted a Continuous Performance Test (CPT) version, a paradigm that can be considered as the gold-standard neuropsychological task in the ADHD assessment^32^. As this is the first EPELI study in an adult ADHD population, we decided to use the measures validated in our studies on children^6,7^ as the main dependent variables.

Based on the few earlier adult ADHD studies indicating problems in complex PM tasks, our first pre-registered hypothesis was that at the group level, the ADHD group shows poorer performance on EPELI than the controls. The second hypothesis was that even at the individual level, EPELI is useful in differentiating between ADHD and neurotypical individuals. Regarding CPT, we included mean reaction time and its standard deviation as dependent variables^33^. To examine whether ADHD is associated with a higher number of attentional lapses during EPELI performance, we conducted an intra-individual variability (IIV) analysis of response latencies (RLs) in EPELI, employing similar methods that have been previously used in CPT studies^33^. As we did not know beforehand how many female participants with ADHD would sign up for the study, we did not pre-register separate analyses related to possible gender differences. However, as an unexpectedly large proportion of females participated in the study, we included a post-hoc analysis examining possible sex effects. This was motivated by the fact that gender differences are known to play a major role in ADHD^17^ but these differences are still not well understood in the adult population.

## Materials and methods

### Participants

The final sample in this study contained 112 adults with ADHD and 255 neurotypical controls. The inclusion criteria for the control group were normal or corrected-to-normal vision; no color blindness; no neurodevelopmental disorders; no neurological illness that affects the participant’s current life; never diagnosed with severe depression, bipolar disorder, psychosis, or schizophrenia across the lifespan; and no self-reported substance-abuse problem. Further exclusion criteria were set according to the DSM-5 Cross-Cutting Symptom Measure^34^ (CCSM). In the control group, the following exclusion criteria regarding CCSM were used: any degree of suicidality (i.e. any other score than 0 in item 11), and sum scores of 3 or more in the domains of depression, mania, or anxiety (i.e. at most “mild” symptoms, or a response indicating occurrence of the symptom not more than during “several days” over the last 2 weeks were acceptable). In the ADHD group we initially used the same exclusion criteria as in the control group. However, after prescreening 855 ADHD participants, we relaxed the CCSM criteria for the ADHD participants to obtain a sufficient sample size in the ADHD group. The observed amount of comorbid symptoms was not surprising considering the commonness of mood and anxiety symptoms in ADHD adults^15^, with roughly 77% of individuals having one or more comorbid disorders^35^. The updated criteria were: suicidality score less than 2 (i.e. at most slight symptoms), and depression and anxiety scores less than 4 (i.e. at most moderate symptoms). Other criteria remained the same for both groups.

The ADHD group was defined as those responding positively to a question probing whether they have been diagnosed by a licensed medical professional as having ADHD or ADD. Additionally, in the ADHD group we only included participants whose responses in the Adult ADHD Self-Report Scale (ASRS-v1.1) Symptom Checklist (part A)^36^ indicated symptoms consistent with ADHD, based on the instructions of the questionnaire (i.e. at least four responses of the participant were in the risk area as defined in the form).

Before further exclusions, the sample size was 293 in the control group and 121 in the ADHD group. Next, we removed all participants who reported that they had cheated or were intoxicated in any of the sessions. This resulted in deleting 8 participants (6.6%) in the ADHD group and 32 participants (11%) in the control group. After this deletion, *n* in the control group was 261 and in the ADHD group 113. Additionally, one participant in the ADHD group and six participants in the control group were removed due to missing background data that was necessary to determine their eligibility in the study, resulting in final *n* = 112 in the ADHD group and final *n* = 255 in the control group. There was some missing data in this final dataset: Eight participants in the ADHD group and 11 in the control group were missing EPELI data due to technical problems and 34 participants in the ADHD group and 45 participants in the control group dropped out after the first testing session. Outlier exclusions based on task performance are described in the section “Outliers” in the Supplementary materials, methods, and results.

The participants were recruited on the crowdsourcing site Prolific. We targeted participants of age 18–50, currently living in the UK, whose first language was English. The data was gathered between August and December, 2021 (see Supplementary materials, methods, and results for prescreening studies (N = 14,443 and N = 2374, respectively).

### Procedure

Altogether 112 participants with ADHD and 255 controls took part in the five main testing sessions. All tasks and questionnaires were counterbalanced between the participants, except for EPELI which was always administered in the first session and the diary questions which were administered in every session. All sessions were performed on separate weekdays and the whole experiment was required to be completed within 14 days. Duration of each session was about 40 min. The participants received £16.67 compensation for taking part in the study if they completed all sessions. If they did not complete all sessions, no compensation was given. Only those participants who completed the first session were allowed to partake in the other sessions; this was done to avoid missingness in the EPELI data.

The participants started the experiment by logging in to the in-house SOILE online testing platform^37^. SOILE was used to gather all other data except EPELI, which is an Unity game developed by Peili Vision LTD (www.peilivision.fi). The participants were redirected from the SOILE testing platform to the EPELI game server by clicking on a link, and the game was run in a separate browser window. After conducting EPELI, the participants went back to SOILE and were asked to fill out the Virtual Reality Sickness Questionnaire (VRSQ)^38^ and a shortened version of the Presence Questionnaire^39^, screening possible side-effects and sense of presence, respectively.

The browser-based EPELI game begins with a guided hearing threshold and volume adjustment, after which the participant conducts a practice session supervised by a virtual character, ‘Vincent’^40^. Field of view in the game is controlled by a mouse. The participant navigates in the apartment by teleporting, which takes place by directing a crosshair at the center of the screen on white spherical hotspots on the ground and clicking the left mouse button. A clock with running time (reset at the beginning of each block) appears in the lower right corner when the participant clicks the right mouse button. Objects can be grabbed and laid on surfaces by clicking on them.

The adult version of the EPELI task consists of 10 blocks. In the beginning of each block, Vincent first orally introduces the theme of the block and then gives a list of 7–8 everyday tasks to be conducted in the virtual apartment. Besides standard tasks (seven per block), five time-based tasks and five event-based tasks were included. The ongoing event-based task was to take the teddy bear to the sofa if it appears to the visual field. This instruction was given only once before the game execution started.

Following the earlier child version of EPELI^7,41^, the main dependent variables were: (1) Total score (correctly performed subtasks), (2) Task efficacy (percentage of relevant actions out of all actions excluding clicks on the waypoints that enable moving around in the environment; ‘relevant’ means actions that contribute to completing the given tasks), (3) Navigation efficacy (total score divided by covered distance), (4) Controller motion (here the controller is computer mouse), (5) Actions (total number of both task-relevant and -irrelevant clicks), (6) Time-based subtask score, (7) Number of clock checks (for the blocks with a time-based subtask), and (8) Event-based subtask score.

After each block, the participants responded to the following open question: “Please describe in as much detail as possible how you solved the previous set of tasks”. This was included to identify strategy employment. Based on our earlier work on coding open-ended memory strategy reports^42–45^, as well as our expectations concerning PM-related strategies, a coding scheme was devised (see Laine et al. in press, for details^46^). Each open-ended response for each of the 10 EPELI task blocks was coded by two independent raters. The raters coded each strategy report on three variables: the first reported strategy type (no strategy, rehearsal/repetition, grouping, action schema utilization, association, visualization, condensing information, selective focus, external cueing, other strategy), the total number of strategy types reported, and the total number of specific strategy details given. Here we focused on the first variable, the primary strategy type, that was dichotomised as strategy use vs. non-use in the analyses.

In addition to EPELI, a battery of other computerized tasks was administered (see Supplementary materials, methods, and results for details). These included, (1) two conventional PM tasks, the Cruiser and Matching tasks; (2) the International Cognitive Ability Resource with 16 items^47^ (ICAR16) tapping participants’ general cognitive ability; (3) CPT^48^; (4) instruction recall task (IR) that tapped episodic memory processes similar to those required in EPELI but without action execution that characterizes EPELI performance^7^; and an episodic memory task called Word List Learning (WLL)^45^. After each task, the participants also rated the verisimilitude of the task, i.e. its likeness to real life (“How much did the task resemble your everyday life?”), answered on a seven-point scale. In addition, they rated task difficulty and their motivation, both answered on a five-point scale. During the whole measurement period with five sessions in different days, the participants also kept an everyday PM diary that included six questions (see Supplementary materials, methods, and results)^23^.

### Statistical analysis

In addition to frequentist p-values, Bayes factors (BF) were calculated for each analysis when applicable (see Supplementary materials, methods, and results for statistical details, as well as final samples and exclusions). Receiver Operating Characteristic (ROC) curve analysis was done in Python with scikit-learn package v1.0.2^49^. A logistic regression classifier was trained to investigate discriminability between participants with ADHD and control participants based on EPELI and CPT data. For IIV analysis, RLs in EPELI were defined as the time interval between two consecutive mouse clicks (for more detail see Supplementary materials, methods, and results). Ex-Gaussian modelling was used^50^ to separate extremely slow responses presumed to be associated with attentional lapses. The ex-Gaussian model assumes that a positively skewed RL distribution can be represented as a mixture of an exponential component (defined by the exponential decay constant – *tau*) and gaussian distribution (defined by the mean – *mu*, and the standard deviation – *sigma*). Strategy use across the two groups and their associations with task performance were examined with Bayesian Linear Mixed Effects Models (LME) using R version 4.0.0 with the “BayesFactor” package^51^. This analysis was only performed for the dependent variables Total score, Navigation efficacy, and Task efficacy. The action-related variables were ignored because actions other than those resulting in correct performance were not expected to be directly related to the memory strategies. Moreover, time- and event-based variables were ignored, as these tasks did not provide blockwise data which is required for the LME analysis.

### Ethical approval

The study was approved by the Ethics Board of the Departments of Psychology and Logopedics at the Åbo Akademi University, Finland. Informed consent was obtained from each participant and all methods were performed in accordance with the relevant guidelines and regulations (including the declaration of Helsinki and the General Data Protection Regulation).

## Results

### Background characteristics

Characteristics of the participants can be found from Table 1. In the ADHD group, the participants reported having their ADHD or ADD diagnosis at the average age of 17.48 (SD = 11.56, range 1–47). 51 had received diagnosis in childhood (< 18 years of age) and 50 in adulthood (≥ 18 years; data was missing for 11 participants). In the ADHD group, 25 (22%) reported using stimulant medication on at least one of the testing sessions and average time since the last dose was 11.53 h (SD = 5.92). As this is a remote study conducted in a different country, we could not have full control over the use of medication and decided to keep these participants in the analyses.

**Table 1:** Background characteristics of the study groups.

	ADHD		Control		U	p	BF_10_
	M	SD	M	SD			
Age	30.34	7.77	31.80	8.71	13,021	0.18	0.15
Education level	5.23	1.62	5.46	1.42	13,008	0.38	0.16
Gaming, EPELI-like	0.36	0.48	0.43	0.50	7620	0.29	0.20
Gaming, hours per week	5.57	6.37	3.69	6.96	10,623	< 0.001***	5.11
ICAR-16, % correct	0.45	0.24	0.50	0.22	7312	0.08	0.29
CCSM, depression average	1.61	0.87	1.08	0.71	19,171	< 0.001***	> 100
CCSM, anxiety average	1.62	0.96	0.97	0.70	19,901	< 0.001***	> 100
Audit sum	3.77	2.55	3.48	2.51	15,233	0.31	0.19

Gender	n	%	n	%	χ^2^	p	BF_10_
Male	37	33%	70	27%	3.57	0.17	0.02
Female	74	66%	185	73%	–	–	–
Other	1	1%	0	0%	–	–	–

*ICAR-16* the international cognitive ability resource, 16 items; *CCSM* DSM-5 cross-cutting symptom measure; *Audit* alcohol use disorders identification test. Asterisks indicate significant differences: *p ≤ 0.05, **p ≤ 0.01, ***p ≤ 0.001.

### Self-rating questionnaires of attention, executive functions, and PM

Participants with ADHD reported more problems in attentional executive functions than neurotypical controls in all questionnaires, except MPMI (Table 2). Evidence for the group differences was overall decisive and the effect sizes were large.

**Table 2:** Self-rating questionnaires of attention, executive functions, and prospective memory.

	ADHD		Control		Cohen’s d	t	p	BF_10_
	M	SD	M	SD				
ASRS, average	2.92	0.45	1.68	0.64	2.24	18.52	< 0.001***	> 100
PRMQ, prospective	3.50	0.87	2.44	0.72	1.32	12.11	< 0.001***	> 100
PRMQ, retrospective	2.96	0.80	2.13	0.67	1.12	10.24	< 0.001***	> 100
ADEXI, average	3.34	0.55	2.52	0.52	1.53	11.84	< 0.001***	> 100
ADEXI, inhibition	3.40	0.71	2.49	0.56	1.42	11.51	< 0.001***	> 100
ADEXI, working memory	3.31	0.62	2.54	0.61	1.26	9.63	< 0.001***	> 100
MPMI, PM ability	3.20	0.44	3.22	0.38	− 0.04	− 0.35	0.73	0.15
MPMI, PM strategy: external	2.32	0.78	2.39	0.72	− 0.10	− 0.75	0.45	0.19
MPMI, PM strategy: internal	2.71	0.80	2.60	0.75	0.13	1.05	0.30	0.24
Diary, average	11.22	3.28	7.59	1.73	1.39	12.07	< 0.001***	> 100

*ASRS* adult ADHD self-report scale, *PRMQ* prospective-retrospective memory questionnaire, *ADEXI* adult executive functioning inventory, *MPMI* metacognitive prospective memory inventory, *PM* prospective memory. Asterisks indicate significant differences: *p ≤ 0.05, **p ≤ 0.01, ***p ≤ 0.001.

### Group differences on EPELI variables

We first examined possible group differences in the eight pre-registered EPELI variables (Table 3). In terms of BFs, there was evidence that participants with ADHD made more Actions (BF_10_ = 10.50). Frequentist t-tests showed worse performance in participants with ADHD also in Task Efficacy (p = 0.015) and Navigation Efficacy (p = 0.047), although Bayesian evidence for these group differences was only anecdotal. As a post hoc analysis, we also examined how performance in these variables that showed group difference evolved over time; there were no Group × Block interactions (p > 0.05; see Fig. S1 in Supplementary methods, materials, and results).

**Table 3:** Task performance of the study groups in the EPELI game.

	ADHD				Control				Cohen’s d	t	p	BF_10_
	M	SD	CI lower	CI upper	M	SD	CI lower	CI upper				
Planned variables												
Total score	55.85	12.04	53.50	58.21	55.62	11.34	54.19	57.05	0.02	0.17	0.862	0.13
Task efficacy	0.34	0.12	0.31	0.36	0.37	0.11	0.36	0.38	− 0.28	− 2.45	0.015*	2.21
Navigation efficacy	0.05	0.01	0.05	0.05	0.05	0.01	0.05	0.05	− 0.24	− 1.99	0.047*	0.85
Controller motion	48,908.92	13,595.79	46,264.88	51,552.96	48,976.73	12,709.65	47,367.35	50,586.12	− 0.01	− 0.05	0.964	0.13
Actions	534.62	170.86	501.23	568.01	484.32	123.89	468.46	500.17	0.34	3.05	0.002**	10.50
Correct event-based tasks	3.22	1.91	2.85	3.59	2.93	2.03	2.67	3.19	0.15	1.25	0.214	0.27
Correct time-based tasks	4.31	0.69	4.18	4.45	4.23	0.79	4.13	4.33	0.11	0.90	0.368	0.19
Looked at the watch	21.27	11.79	18.95	23.58	21.68	11.32	20.25	23.12	-0.04	-0.31	0.758	0.14
Post hoc variables												
Total time	2458.33	354.23	2388.75	2527.90	2483.08	373.84	2435.64	2530.52	− 0.07	− 0.57	0.570	0.15
Total movement	678.54	151.21	648.99	708.10	679.04	146.42	660.46	697.62	0.00	− 0.03	0.977	0.13
Winding number	− 0.48	10.12	− 2.45	1.49	1.18	8.80	0.07	2.29	− 0.17	− 1.54	0.126	0.40
Total click distance	657.47	240.23	610.52	704.42	557.10	173.66	534.97	579.23	0.48	4.34	< 0.001***	> 100
Empty clicks	70.81	25.84	65.74	75.89	61.16	17.67	58.91	63.42	0.44	3.99	< 0.001***	> 100
Clicks on doors and drawers	43.95	20.04	40.01	47.89	38.27	17.39	36.04	40.49	0.30	2.63	0.009**	3.47
Relevant clicks	132.39	27.11	127.09	137.69	131.50	27.22	128.06	134.95	0.03	0.28	0.782	0.13
Number of grabbed objects	65.49	19.66	61.64	69.33	56.55	13.93	54.78	58.32	0.52	4.79	< 0.001***	> 100

Asterisks indicate significant differences: *p ≤ 0.05, **p ≤ 0.01, ***p ≤ 0.001.

‘Winding number’ refers to the amount of full rotations of the participant’s field of view. A full rotation clockwise receives the value of 1 and a full rotation counterclockwise-1. The value of the variable is the sum, positive value indicating more clockwise rotations than counterclockwise rotations.

To further interpret the group differences in Actions, we conducted post hoc analyses with other EPELI variables related to different subcategories of Actions. Both Bayesian and frequentist analyses indicated that the participants with ADHD made more empty clicks (e.g. clicks to the walls or floor), clicked more on doors and drawers, grabbed more objects, and their click distance was higher (i.e. they interacted with objects further away) (Table 3).

Due to the group difference in everyday playtime (i.e. the self-reported amount of hours spent playing computer games on average per week; see Table 1), we conducted a separate ANCOVA where this background variable was included as a covariate. In these analyses, there was anecdotal evidence for the effect of Group in Task efficacy (F = 4.39, p = 0.037; BF_10_ = 1.56), but not in Navigation efficacy (F = 3.47, p = 0.064; BF_incl_ = 0.91). In Bayesian terms, these effects are consistent with the analysis where the covariate was not included. However, ANCOVA including average playtime as a covariate did not show evidence for the effect of Group on Actions (F = 2.06, p = 0.15; BF_incl_ = 0.41). As to the post hoc variables, the effects of Group on Total click distance (F = 7.17, p = 0.008; BF_incl_ = 4.67), Empty clicks (F = 13.06, p < 0.001; BF_incl_ > 100), and Number of grabbed objects (F = 13.20, p < 0.001; BF_incl_ = 76) remained. However, there was no longer an effect of Group in Clicks on doors and drawers (F = 0.75, p = 0.39; BF_incl_ = 0.22).

**Figure 1:**
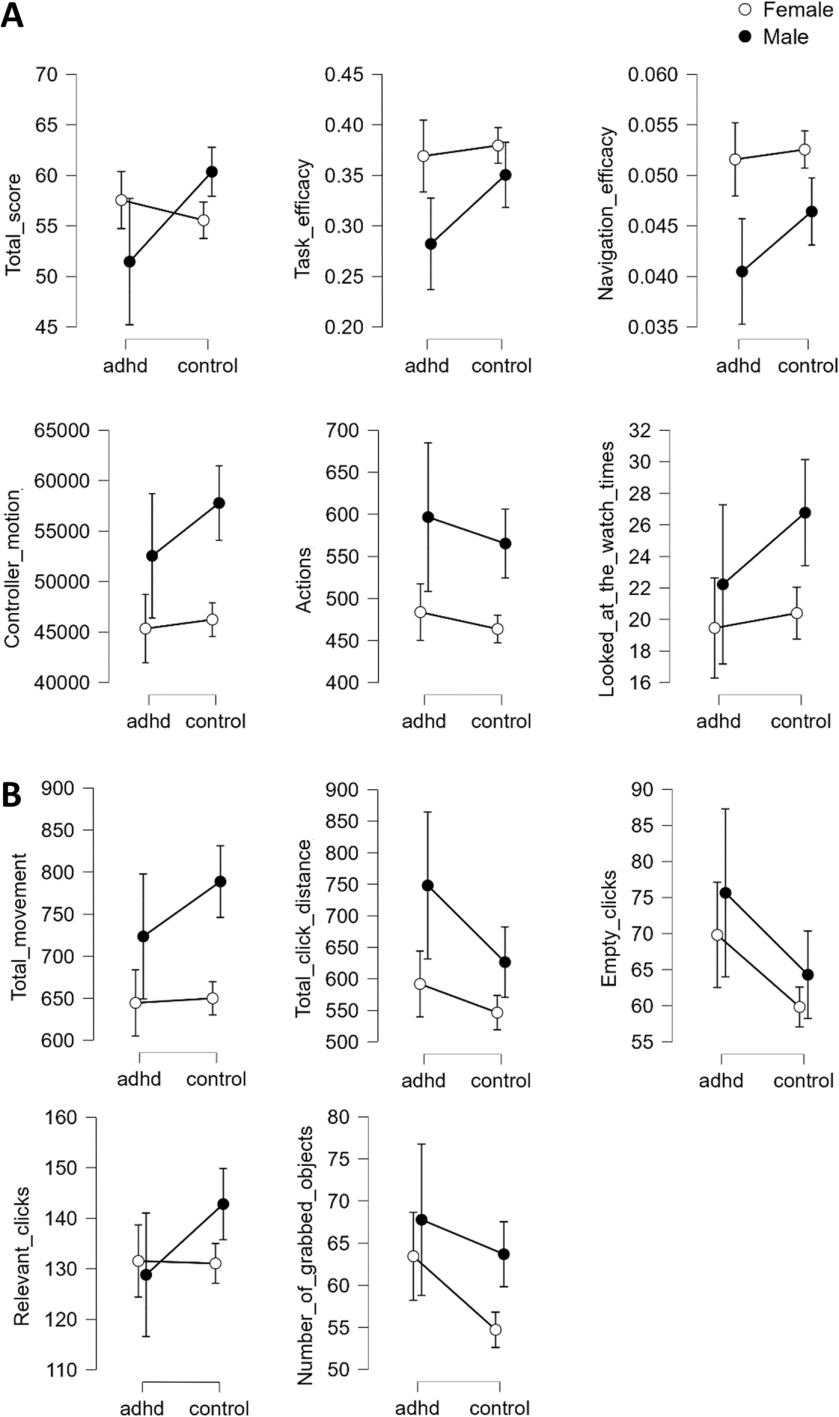
Effects of group and gender, as well as group × gender interaction on EPELI variables with gaming experience as covariate. Only significant effects (p < 0.05) or effects with substantial evidence (BF > 3) are shown.

Next, to examine gender effects we conducted an ANCOVA with Group and Gender as fixed factors and average playtime as a covariate. One participant in the ADHD group who reported “other” as gender was omitted from this analysis due to this being a single observation. The results are summarized in Table 4 and Fig. 1. Overall, in the planned variables males showed lower efficacy and higher amount of actions than females. Moreover, there was evidence of an interaction effect in Total Score, where the group difference was pronounced for males. Regarding the post hoc variables, males were more active than females and manipulated objects from further distance.

**Table 4:** ANCOVA analyses of task performances in the EPELI game with Group (ADHD/control) and gender (male/female) as fixed factors and playtime (average gaming hours per week) as covariate.

	Group			Gender			Interaction		
	F	p	BF_incl_	F	p	BF_incl_	F	p	BF_incl_
Planned variables									
Total score	5.69	0.018*	1.20	1.06	0.304	1.00	11.55	< 0.001***	4.54
Task efficacy	5.38	0.021*	1.01	10.09	0.002**	11.63	3.15	0.077	1.30
Navigation efficacy	3.68	0.056	0.52	20.93	< 0.001***	> 100	2.12	0.147	0.63
Controller motion	4.85	0.028*	0.71	19.89	< 0.001***	> 100	1.69	0.195	0.82
Actions	0.95	0.330	0.19	20.42	< 0.001***	> 100	0.11	0.742	0.16
Correct event-based tasks	0.00	0.962	0.11	0.06	0.810	0.14	0.20	0.658	0.02
Correct time-based tasks	0.09	0.765	0.11	0.21	0.647	0.13	0.76	0.384	0.03
Looked at the watch	3.41	0.066	0.36	5.82	0.017*	17.03	1.26	0.262	0.45
Post hoc variables									
Total time	0.00	0.963	0.11	0.16	0.687	0.12	0.08	0.784	0.02
Total movement	4.46	0.036*	0.54	21.84	< 0.001***	> 100	2.49	0.116	0.88
Winding number	0.91	0.342	0.23	1.15	0.284	0.13	1.07	0.301	0.05
Total click distance	7.11	0.008**	2.62	12.78	< 0.001***	47.81	1.67	0.197	1.21
Empty clicks	11.62	< 0.001***	67.67	1.74	0.188	0.45	0.05	0.818	0.31
Clicks on doors and drawers	0.17	0.683	0.17	3.38	0.067	1.43	0.38	0.541	0.11
Relevant clicks	4.42	0.036*	0.34	0.22	0.637	0.34	3.96	0.048*	0.38
Number of grabbed objects	7.87	0.005**	29.88	6.30	0.013*	24.56	0.95	0.330	1.48

BF_incl_ refers to Bayes factor for the inclusion of the variable, that is, the change from prior to posterior inclusion odds. This variable is interpreted in the same way as BF_10_. Asterisks indicate significant effects: *p ≤ 0.05, **p ≤ 0.01, ***p ≤ 0.001.

We also examined whether age was associated with EPELI performance. There was strong evidence that age correlated negatively with Total score (r = − 0.28, p < 0.001, BF_10_ > 100) and Correct time-based tasks (r = − 0.23, p < 0.001, BF_10_ > 100), and with several variables related to interaction with the game environment. All correlations between age and EPELI variables are reported in Appendix A.

A logistic regression classifier using the pre-registered EPELI variables showing significant differences between the groups (Total Actions, Task Efficacy, and Navigation Efficacy) as a predictor, resulted in 0.61 Area Under the Curve (AUC) score that was higher than for randomly labeled permutations (t(58) = 10.17, p < 0.0001). Similar classifiers trained separately for each gender yielded AUC score of 0.64 for male data, and AUC score of 0.58 for female data. The gender difference in the classifier performance was significant (t(58) = 7.47, p < 0.0001). A classifier trained using variables related to subcategories of Total Actions (empty clicks, clicks doors and drawers, relevant clicks, number of grabbed objects) provided higher performance (t(58) = 14.63, p < 0.0001) with an AUC score of 0.65. Analysis of feature importance showed that the same classification accuracy can be achieved with reducing the features to two variables: relevant clicks, and number of grabbed objects. Training on the data separated by gender resulted in AUC = 0.67 for males, and AUC = 0.65 for females. There was no difference in performance between classifiers with male and female data here.

IIV analysis included 162 089 observations, with mean RL in the clinical group 1.94 s (± 1.02 s), and in the control group 2.06 s (± 1.1 s). The ex-Gaussian analysis revealed that mu was shorter for ADHD participants than for controls across all clicks and for irrelevant clicks. There were no group differences in sigma or tau across all clicks or for relevant and irrelevant clicks analyzed separately (see Supplementary materials, methods, and results for more details).

In the ADHD and the control group, spontaneous strategy usage was deployed in 39.1% and 44.8%, respectively, across all the blocks. Strategy use did not differ between the groups (t = 1.28, p = 0.20, BF_10_ = 0.28). To examine associations between strategy use on performance across the groups, we specified LME models with the outcome variable of interest as dependent variable. Group (ADHD/controls), Strategy use (yes/no), and their mutual interaction served as fixed effects, and participant and block as random effects. There was very strong evidence for a main effect of Strategy on Total score (Mdiff = − 0.55 95% HDI [− 0.62 – − 0.49] BF_10_ > 100), Navigation efficacy (Mdiff =  −  0.01 95% HDI [− 0.01 – − 0.00] BF_10_ > 100), and Task efficacy (Mdiff = − 0.05 95% HDI [− 0.06 – − 0.04] BF_10_ > 100), suggesting that those participants using strategies performed better on these three performance measures, as compared to those not using strategies. However, the BF indicated evidence against an interaction effect between Group and Strategy on Total score (Mdiff = 0.01 95% HDI [− 0.05 – 0.07] BF_10_ = 1/16.67), Navigation efficacy (Mdiff = 0.00 95% HDI [0.00 – 0.00] BF_10_ = 1/16.67), and Task efficacy (Mdiff = − 0.01 95% HDI [− 0.01 – 0.00] BF_10_ = 1/14.29), indicating that there was no difference in how strategy use was associated with performance across the two groups.

### Task performance in other tasks

Next, we examined group differences in the other performance-based tasks besides EPELI. Participants with ADHD had higher reaction time variability and omission rate in the CPT task, as well as lower accuracy in the word recall task (Table 5). No evidence of group differences in the other tasks examined here was found.

**Table 5:** Performance of the study groups in the other cognitive tasks (for details of matching, cruiser, instruction recall, and word list learning, please see Supplementary materials, methods, and results).

	ADHD				Control				Cohen’s d	t	p	BF_10_
	M	SD	CI lower	CI upper	M	SD	CI lower	CI upper				
Cruiser, time-based variant: refills	3.36	1.61	2.97	3.76	3.45	1.52	3.23	3.66	− 0.05	− 0.38	0.708	0.17
Cruiser, time-based variant: fuel checks	30.41	22.78	25.34	35.48	26.29	12.59	24.58	28.01	0.26	1.95	0.052	0.86
Cruiser, event-based variant: refills	3.09	1.49	2.69	3.48	3.50	1.34	3.29	3.71	− 0.30	− 1.93	0.054	0.93
Matching, time-based variant, hits	2.87	1.14	2.54	3.21	2.83	1.33	2.61	3.04	0.04	0.21	0.836	0.18
Matching, event-based variant, hits	3.00	1.35	2.64	3.36	3.40	1.34	3.20	3.61	− 0.30	− 1.94	0.053	0.95
CPT commission errors	17.21	6.76	15.71	18.72	15.56	6.24	14.71	16.41	0.26	1.97	0.049*	0.89
CPT reaction time variance	138.67	81.86	120.33	157.00	97.94	43.48	91.98	103.90	0.72	5.43	< 0.001***	> 100
CPT omission errors	9.27	14.10	6.07	12.47	4.59	8.27	3.46	5.72	0.46	3.45	< 0.001***	36.82
Instruction recall, correct items	3.71	1.78	3.33	4.09	4.06	1.61	3.85	4.28	− 0.21	− 1.66	0.097	0.52
Word list learning, correct items	10.55	3.36	9.81	11.29	11.74	2.70	11.38	12.11	− 0.41	− 3.15	0.002**	14.55

*CPT* Conner’s continuous performance task. Asterisks indicate significant differences: *p ≤ 0.05, **p ≤ 0.01, ***p ≤ 0.001.

Due to the group difference in CPT, we trained a classifier using related RT variability to further examine how well this measure is able to predict the group status of individual participants. The AUC score obtained based on this classifier was 0.68, which is higher than the one obtained for combined EPELI data (t(58) = 15.35, p < 0.0001). When the classifier with CPT data was conducted separately for male and female data, AUCs were 0.65 and 0.71, respectively. The difference between these two classifiers is statistically significant (t (58) = − 15.39, p < 0.0001).

To shed further light on how EPELI and CPT are related to everyday ADHD symptoms, we examined the associations between the self-rated symptoms (ASRS-A; the Diary for prospective memory failures), the EPELI variables, and CPT. The intercorrelations were calculated for the pooled data, and for the ADHD group only. The correlations are shown in Appendix B.

The pooled data indicated that ASRS-A score was positively associated with EPELI Total actions, Click distance, Empty clicks and Number of grabbed objects (p < 0.05; BF_10_ = 3.48 – 11.61). In the same vein, ASRS-A score was positively correlated with CPT Commissions (p = 0.006; BF_10_ = 3.00) and Mean SD (p < 0.001; BF_10_ = 70.71). Regarding the Diary score and EPELI interrelationships, there was evidence for a positive correlation with Empty clicks (p = 0.001; BF_10_ = 15.36), and anecdotal evidence for Total actions, Click distance, Number of grabbed objects, and Total time (p < 0.05; BF_10_ = 1.12 – 2.53). The Diary score showed strong evidence for a correlation with CPT Mean SD (p < 0.001; BF_10_ > 100), and anecdotal evidence for a correlation with CPT omissions (p = 0.022; BF_10_ = 1.03).

Within the considerably smaller ADHD group, some of the correlations listed above were observed, but only with anecdotal evidence. ASRS-A was associated with CPT Commissions (p = 0.039; BF_10_ = 1.13). The Diary score correlated with Controller motion, Total actions, Total time and Clicks on doors and drawers (p < 0.05; BF_10_ = 1.03 – 1.94).

## Discussion

We conducted a large-scale online study examining goal-directed behavior in adults with ADHD using a new EPELI game that simulates everyday life PM conditions with high attentional executive demands. We carefully screened 14 443 participants with a Prolific crowdsourcing platform and identified 337 eligible candidates among 2374 adults with ADHD of whom 112 took part in the study. Based on earlier PM studies in adults with ADHD^26,27,52^ and our results in children with ADHD using a largely similar paradigm to the one employed here^7^, we hypothesized that participants with ADHD will show poorer performance in EPELI as compared with neurotypical controls. However, robust evidence of differences between ADHD participants and controls was found in only one out of the five main EPELI measures that showed group differences in previous child studies, and not in the two additional PM tasks (i.e. Cruiser and Matching) included in this study. The only EPELI main measure showing clear evidence for a group difference was the number of actions conducted during the game play, which was higher for participants in the ADHD group. However, after controlling for everyday video gaming frequency where group differences were observed, the difference remained significant only in specific subtypes of actions (i.e. empty clicks and how many objects the participant grabbed in the game). In this analysis, two other measures besides actions, namely Task efficacy (i.e. percentage of relevant actions out of all actions), and Navigation efficacy (i.e. Total score divided by distance covered), also showed modest group differences. Actions and other related EPELI variables, as well as several CPT variables, were also correlated with self-reported ADHD symptoms across the whole sample. To a less extent, this was true also for correlations with the Diary summative score for everyday prospective memory failures.

The gender distribution among ADHD participants who registered for the study was not representative of the general population of people with ADHD where the ratio is about 3:1 of men to women^53^. Also considering the strong evidence that the symptoms^16,17^ and cognitive performance^54^ differ between female and male adults with ADHD, post hoc analyses with gender as a factor were conducted. As expected, this analysis revealed more prominent group differences in men than in women in the EPELI Total score. Main effects of gender were also observed in several other variables, for example, Task Efficacy, Navigation Efficacy, Actions, and Controller motion.

Taken together, this study indicates that the present, new online version of EPELI is feasible to use with adults, and that especially certain aspects of actions taken in the game can differentiate adults with ADHD from neurotypical controls. At the same time, the group differences and correlations with subjectively reported symptoms in this particular prescreened sample were more limited than in earlier EPELI studies in children. We discuss these results in more detail below.

### Group differences between ADHD adults and neurotypical controls

Consistent with the two studies conducted in children^6,7^, we observed that ADHD participants were overall more active in interacting with the virtual environment than controls. In previous studies, we have interpreted higher numbers of actions in ADHD participants to reflect impulsive behavior associated with irrelevant objects, as in the child version much of these extraneous actions relate to interacting with attractive objects (e.g. toys, instruments or devices like TV). Impulsive behavior is a characteristic for ADHD, not only in children but also in adults^55^. Regarding types of irrelevant actions, we found that ADHD participants in the present study especially picked up irrelevant objects more frequently but showed also irrelevant actions in opening doors and drawers and in button presses to areas where there were no objects (e.g. empty parts of the floor and walls). In a previous child study with VR goggles, we recorded the gameplay videos and further labeled the actions based on the object salience. However, the group differences appeared not to be explained by stimulus-driven capture of attention^6^. The present findings align with this, as there were group differences also related to exploratory actions that do not specifically relate to object interaction. Participants with ADHD also interacted with the objects from longer distances. As ADHD participants played video games more often in their free time and this factor was explaining group differences in Total actions, these results should be interpreted with caution. It should be noted that the association between playtime and its link to EPELI performance is not entirely straightforward to interpret, as there is evidence that gaming may influence cognitive functions^56^, and there is previous evidence that ADHD symptoms are associated with gaming^57^. Nevertheless, the playtime variable explained part of the group differences and only the subcategories of actions continued to show group differences after accounting for participants’ playtime.

Together our findings related to atypical interaction with the environment indicates less efficient behavior that did not result in poorer task performance. Hence, it is not likely to reflect cognitive capacity as such. In EPELI, both the targets of actions and frequency of actions relate to volitional behaviors that participants initiate to interact with an open-ended environment. While the operationalization of impulsivity has been under active discussion during the past decades^55^, the opportunities for implementing impulsivity measures reflecting unusual interaction with life-like situations have not been examined previously in adult ADHD. Hence, despite the present rather modest group differences, new paradigms like EPELI provide ways to operationalize new ecologically valid measures for quantifying behavior in complex situations. Examining spontaneous behavior in an open-ended environment with high cognitive demands may thereby open a new avenue to detect deviant features in typical behavior in adults with ADHD that has been understudied so far^5^.

In our child studies, Task efficacy has been the most sensitive measure in differentiating ADHD participants from controls^6,7^. As Task efficacy is a ratio between total score and all actions and as there was no group effect in total score, this difference mainly comes from differences in task-irrelevant behavior. This could be the situation also regarding the Navigation efficacy, another relative measure of EPELI. Such deviant way of interaction when moving around in the apartment is further evidenced by the group differences in the average click distance. Since this is the first study in adults with ADHD where a naturalistic PM task was used, a direct comparison to previous findings cannot be established. However, considering the prior studies in ADHD adults indicating that even at group level systematic differences between ADHD participants and neurotypical controls are rarely observed^12^, it is not surprising that the group differences were small also in this sample. Findings in this sample reveal some potential mediating factors that should be accounted for when analyzing real-world goal-directed behavior in ADHD adults. Such factors include at least the use of strategies that ADHD adults may have acquired to compensate for their challenges^20^ and the effect of gender and age. More research on these factors that modulate group differences is needed. Due to the complex effects of age, gender, gaming background and other factors that are likely to be intertwined, larger samples are needed to tease apart these effects. A larger dataset or some other type of strategy manipulation (e.g. externally given strategies), would also be needed to obtain enough observations per task block to probe the efficacy of specific strategy types in ADHD. Meanwhile, a separate study on strategy employment by the much larger control group of ours^46^ identified Grouping as a particularly effective specific strategy for EPELI.

With regard to measuring hyperactivity, our child studies used the movement of a hand controller instead of mouse movement. It is possible that the hand controller measuring movement of the whole hand when it is moving freely could be a more sensitive indicator of hyperactivity than mouse movement that is more restricted, rigid and probably also overlearned. Alternatively, it could be that hyperactivity is simply more difficult to detect in adulthood. Hyperactivity is typically the symptom domain where the spontaneous remediation across the development is largest^8^. Consistent with these findings, Controller motion was negatively correlated with age in the present sample. It could be that such hyperactivity effects can be only observed in special population and perhaps with particularly sensitive methods such as sensor-based motion tracking of excessive activity levels in daily life over several days^58^.

Regarding the classifiers, EPELI’s performance was clearly lower in this adult sample than in previous EPELI studies in children^6,7^, which was expected based on the limited group differences in EPELI performance. This was the case also for CPT and the performance in EPELI and CPT were correlated, suggesting that the two tests may measure partially the same construct.

To further compare EPELI and CPT, we also included RL measures including IIV. However, unlike in the CPT where standard deviation of RLs was higher in the ADHD group as compared with the control group, we did not find evidence of such a difference suggesting fluctuation of sustained attention in EPELI. In children, we have preliminary evidence of higher IIV in the ADHD group than in the control group^59^. Consistent with our findings in children, our present results did provide some evidence that ADHD adults may, however, be faster in their interaction with the environment than control participants. The differences between EPELI and CPT in RLs and also other measures could be partially related to the continuously changing nature of the EPELI tasks. This may help individuals with ADHD to attend better to EPELI than to CPT^60^. Due to their different properties (e.g. open-ended vs. prestructured, self-paced *vs.* externally-paced, dynamic vs*.* static) these two tasks may have complementary features.

In general, considering the previous literature these classification rates were not unexpectedly low^33^ and hence we can conclude that adult ADHD is simply a challenging target for quantitative behavioral assessment^12,61^. Despite the need for further examining the metrics that effectively differentiate adults with ADHD from neurotypical adults, this study provides important evidence demonstrating that online platforms for automatized neuropsychological assessment have high potential and currently remain vastly underinvestigated as a means to support clinical assessments. Unlike the child version that is currently already taken into clinical use in Finland, the present results do not yet provide clear evidence that the online version of EPELI could identify consistent behavioral problems in ADHD adults at the level that would be sufficient for diagnostic purposes. However, as the reliability and validity (verisimilitude) of the test is reasonable^31^, the broad neuropsychological characterization that EPELI provides could possibly be useful for other clinical purposes, similar to many other tests of executive functions that are not able to detect the presence vs. absence of ADHD in individual participants. Especially in crisis situations like COVID-19 where regular clinical assessments are difficult to perform, or when there are other practical or resource related issues hampering conventional examination, home-based assessment could be considered. Possibly the clinical applicability of EPELI could be further improved by including other metrics such as eye tracking, which we have also used in a child study^6^. This could provide more information of the group differences and particularly those related to inattention for which we did not here find as clear evidence as for impulsivity.

### The effect of gender on EPELI performance and observed group differences

Gender differences in the symptoms have been a key topic in recent ADHD research, as their existence could considerably influence the accuracy of detecting ADHD in women as well as increase the risk for misdiagnosis^17^. In keeping with these clinical findings and our previous study in children, clear gender differences in EPELI were observed. However, the gender differences explained group differences only in the Total score where female participants performed on average better than the male participants. Also in children, we have observed gender differences favoring female participants in the Total score^41^. The other main EPELI measures for which a main effect of gender was observed in this study were Task efficacy, Navigation efficacy, Total actions, and Controller motion. Out of these measures, gender differences in children were observed in all other measures, except Controller motion. However, unlike in the present study, we have not yet been able to analyze interactions between gender and group in children, as the sample sizes have been too small for this purpose. Hence, this is the first study reporting how gender may influence the detection of group differences when measuring goal-directed behavior and prospective memory in an open-ended task simulating real-world situations.

Why gender differences were observed also in other measures than Total score goes beyond this study. To further interpret such effects, it would be useful to go deeper into the background measures and first see if some mediating factors may cause these effects (e.g. education, IQ, psychiatric symptoms). For such purposes, the ADHD sample reported here is rather small. With regard to the gender differences within the neurotypical sample, multivariate analyses such as structural equation model would be possible to conduct in a separate study, and the background data collected here at the prescreening stage provides opportunities to further examine the gender differences in ADHD with respect to the symptoms and self-rated attentional-executive problems. One open question regarding the gender differences is also why women with ADHD appeared to be clearly more active in signing up for this study than men, especially considering the gender-specific prevalence rates^11^. We can only speculate whether this was due to some very practical reason (e.g. information spreading in particular social media channels), indicates women being generally more active in participating to this type of studies (female bias was observed also in the control group), or if women with ADHD are somehow more interested to learn from their condition by experiencing how the related problems can be assessed. Whatever the reason, the non-representative gender distribution should be carefully considered when interpreting our results. In case the group of ADHD females that signed up for the study was somehow not representative of the general population, it could also influence the group differences. However, based on the present findings there is no clear evidence of that (e.g. the self-reported symptoms were prominent in this ADHD population).

### Limitations of the study

There are some limitations that should be considered when interpreting the results. First, although the sample size is reasonable, it is far from being representative of the population, as less than 10% of the ADHD adults who took part to the first prescreening ended up in this sample (of those passing the first prescreen, 14% were eligible after screening the comorbid symptoms in the second prescreen). The present participants represent the minority of ADHD population who have minimal other symptoms. In this narrow population, a clear majority of adults with ADHD appeared to be females. Rare use of medication could also hint that the present population represents those with mild symptoms. It is possible that online data collection could involve some other limitations too, such as uncontrolled measurement situation, or even cheating. However, the participants had no incentive not to report if they cheated and if they did, they were omitted from the sample.

## Conclusions

This study showed that online cognitive testing with a 3D videogame is a feasible way to assess variety of attentional executive functions in adults with ADHD. However, in the present sample with minimal rates of other symptoms (representing about 8% of the originally prescreened adults with ADHD and about 0.8% of the full prescreened population), the group differences were quite limited both in EPELI as well as in other tests included in the test battery. In comparison to the previous child studies where the eligibility was not tested at the level of single symptom items, as it rarely happens in clinical studies, another clear difference was that the gender distribution was not representative of the ADHD population, with several times more females than would be expected. These three factors together – studying the minority of individuals with ADHD who report minimal number of other problems, targeting adults whose symptoms are generally milder than those of children, and sampling mostly females whose symptoms are more difficult to detect than in males—make a difficult combination for any test. At the same time, the present observations in this population give important information, as objective detection of ADHD in adults is generally an unmet challenge. What is probably most challenging is to be able to differentiate ADHD symptoms from concomitant symptoms of mood or anxiety disorders, and other psychiatric symptoms.

Even considering these circumstances, some group differences were found. In EPELI, the group differences were mostly observed as unusually high number of task-irrelevant actions, mostly related to unnecessary interaction with the objects, opening doors or drawers to check what is found, and interacting over higher distances to manage the tasks. Such objective description of spontaneous behavior in open-ended lifelike situation brings a valuable new contribution to the field. While the cognitive capacity in our sample was apparently high, there were clear qualitative differences in how the individuals interacted with the virtual environment. More work is still to be done to fully understand the complex factors influencing the detection of ADHD adults with 3D games such as EPELI, as clear gender differences and a moderating effect of playtime were observed. There are various untested opportunities in developing the sensitivity and specificity of EPELI in detecting ADHD in adults. There are other possible factors that could be measured with similar tasks, for example, the role of the available reward or distractibility^6^. Also more powerful data analysis methods, such as machine learning, employed with the rich data could be helpful^58^, especially in attempts to differentiate multiple covarying background factors. Considering the clinical applications, one potential use of EPELI could be in monitoring of the treatment effects (e.g. objective evaluation of the efficacy of stimulant treatment). However, more research is still needed to demonstrate test–retest reliability of EPELI and to account for the possible learning effects reported in our recent study^62^. Such learning effects could be circumvented, for instance, by creating parallel versions that are equally demanding. Altogether, this initial online video gaming study opens interesting avenues for further research and development of clinical assessment methods.

## Supplementary Information

Supplementary Information 1.

Supplementary Information 2.

Supplementary Information 3.

## Data Availability

The datasets generated and analyzed during the current study are available in the Open Science Framework repository, https://osf.io/3y5je/.
