# Supplementary material for: Assessment of goal-directed behavior and prospective memory in adult ADHD with an online 3D videogame simulating everyday tasks
Source: Sci Rep. 2023 Jun 8;13:9299. doi: 10.1038/s41598-023-36351-6 (PMC10248336; doi:10.1038/s41598-023-36351-6)
Supplement: Supplementary file 3 — Supplementary Information 3. [file 41598_2023_36351_MOESM3_ESM.docx]

**Supplementary materials, methods, and results**

*Main article: Assessment of goal-directed behavior and prospective memory in adult ADHD with an online 3D videogame simulating everyday tasks*

**Supplementary materials and methods**

***Prescreenings***

Two prescreening studies (*N* = 14,443 and *N* = 2374, respectively) were first conducted to identify a sufficient number of participants who met the inclusion criteria. The first prescreening study included a question whether the participant had ADHD/ADD diagnoses and the ASRS-A questionnaire to determine the severity of the symptoms (Adler et al., 2006). Out of the 14,443 participants taking part to the first prescreening study, 995 reported having ADHD and 333 had attention deficit disorder (ADD). Of these participants, 89% also satisfied the ASRS criterion (see “Exclusion and inclusion criteria” below) and were invited to the second prescreening, together with control participants who did not report as having ADHD/ADD.

The second prescreening was performed by 2,374 participants, of whom 642 had ADHD, 213 had ADD, and 1,513 were neurotypical. The second prescreening study included more extensive background questionnaires about demographics, mental health, and possible diagnoses that could influence the results. More specifically, the B part of ASRS questionnaire (v1.1) was included to cover the symptoms more thoroughly. DSM-5 Self-Rated Level 1 Cross-Cutting Symptom Measure—Adult (CCSM) (Bravo et al., 2018) was used to assess other domains in mental health. Anxiety subscale in the International Personality Item Pool (IPIP) (Ashton, Lee, & Goldberg, 2007) and the 16-Item Quick Inventory of Depressive Symptomatology (QIDS-SR16) (Rush et al., 2003) were included to gather more detailed information on the possible anxiety and depression symptoms, respectively. After the second prescreening, suitable participants (see the exclusion criteria above) were invited to the actual testing sessions until the minimum sample size of 100 ADHD and 250 neurotypical participants was met.

***Additional cognitive tasks***

In addition to EPELI, two classical prospective memory (PM) tasks were administered. These were Cruiser and Matching, described in detail in Jylkkä et al. (2023). Cruiser is a video game where the participants drive a car on a multilane road in busy traffic, overtaking other cars and trying to avoid hitting them (Kerns, 2000; Voigt, Aberle, Schönfeld, & Kliegel, 2011). The Cruiser has a time-based version, where the participants are instructed to refuel when the fuel tank is getting close to being empty. This happens at 60-second intervals and for 5 cycles. In the event-based version, the participants are instructed to refuel when a bed of yellow flowers is seen by the street. The target (yellow flowers) appeared at five pseudo-random times during the whole task. Both time-based and event-based versions last for 5 minutes. The main dependent variable was performance accuracy, defined as the number of correct PM responses and calculated separately for each of the two task variants. Additionally, monitoring (i.e., the number of times that the gauge was checked) was recorded in the time-based task.

The Matching task also includes a time-based and an event-based version embedded in, and an ongoing primary task. Here the ongoing primary task requires the participant to determine if two simultaneously shown colored symbol strings (e.g., " 874G2" and "834G2", displayed with different font colors) are identical or not (irrespective of color). In the time-based variant, the PM task is to be performed whenever a timer shows the last 5 seconds of a given minute. In the event-based variant, the target button is to be pressed each time one of the symbol strings is red and the other one is blue. Both versions contain five targets, and the duration of the tasks is 5 minutes. The accuracy defined as the number of correct PM responses was the dependent variable in both variants. The time-based version was also planned to yield a measure of time-monitoring (i.e., the number of times that the timer was checked), but this measure was missing due to a programming error.

The Conner’s Continuous Performance Task (CPT) was implemented following (Conners, Epstein, Angold, & Klaric, 2003). In this task version, 360 letters are presented, one at a time, for 250 ms. The task consists of 18 consecutive blocks with varying inter-stimulus intervals (ISI) (1, 2, or 4 seconds) that each had 20 trials. The order of the blocks is pseudo-randomised so that all three ISI conditions occur in every three blocks but in a different order. The participant is instructed to press the spacebar when any letter except the letter “X” appears on the screen. The percentage of trials with letters other than “X” was 90% consistently across all blocks. Dependent variables were the number of omission and commission errors, as well as mean reaction time and standard deviation of reaction times.

Instruction recall task (IR) is developed to measure the episodic memory processes similar to the ones required in the EPELI game, without action execution that characterizes EPELI performance. In this immediate recall task, eight written EPELI-like lists of instructions are presented to the participant, one at a time on the screen. Each instruction was shown for four seconds with one second inter-stimulus-interval. The number of correctly recalled instructions written down by the participants was used as the dependent variable.

In addition to IR, another delayed recall episodic memory task was also administered, called Word List Learning (WLL) (Waris, Fellman, Jylkkä, & Laine, 2020). The participants were presented with a list of 18 words that they must recall after an intermediate arithmetic task. Each word was presented for one second, with an inter-stimulus-interval of one second. There were three blocks with the same words presented in random order. The main dependent variable was the average number of words recalled. After each block, the participant reported the strategies they used in that task block; this data is reported separately.

After each task (including EPELI), the participants also rated the verisimilitude of the task, i.e., its likeness to real life (“How much did the task resemble your everyday life?”), answered on a seven-point scale). In addition, they rated task difficulty and their motivation, both answered on a five-point scale.

The International Cognitive Ability Resource with 16 items (ICAR16). This test (Condon & Revelle, 2014) was used to measure participants’ general cognitive ability.

***Outliers***

In the cognitive tests (but not in the questionnaires), we removed as univariate outliers all observations that were three standard deviations (3 SD) away from the group mean. Additionally, in the PM tasks (Cruiser and Matching), the prospective memory task performance (i.e., pressing the target button or refueling) was defined as an outlier if the participant had an extreme (i.e., ≥ 3 SD deviance from the group mean) amount of false alarms (i.e., performances of the PM task outside the target time or when the cue was not present), or if their performance in the main task was an extreme outlier (i.e., ≥3 SD deviation from the group mean in the number of crashes in Cruiser or in the rate of correct responses in Matching), or if they failed to correctly recall what they were supposed to do in the task (this latter criterion is standardly used in PM tasks to make sure that the performance reflects PM performance and not just episodic memory performance).

In the control group, these criteria resulted in the deletion of 14/1895 observations (0.73%) across all the eight EPELI variables, and 8/633 observations (1.3%) over the three CPT variables. As to the Cruiser tasks, we removed 57/212 observations (27%) in the Cruiser event-based PM variable (of which 56 were due to failure to recall instructions), 17/212 observations (8.0%) in the Cruiser time variable “refills” (of which 14 due to failure to recall instructions), and 3/212 observations (1.4%) in the “fuel checks”. In the Matching task versions, we removed 45/214 observations (21%) in Matching event-based variant (of which 43 due to failure to recall instructions), and 63/214 observations (29%) in the Matching time-based variant (of which 58 due to failure to recall instructions). Note that in the Matching time-based variant, time monitoring behavior could not be examined because that data was not saved due to a programming error. Finally, 3/633 observations (0.47%) were removed in the Word List Learning task and none were removed in the Instruction Recall task.

In the ADHD group, these criteria led to the deletion of 5/832 (0.6%) observations over the eight EPELI variables, and 4/240 (1.7%) over the three CPT variables. In the Cruiser event-based PM variable, 23/80 (29%) of all observations were removed, of which 22 due to instruction recall failure. In the Cruiser time-based PM variable, we removed 15/81 (19%) observations, 13 of them due to instruction recall failure; moreover, 1/81 (1.2%) observations were removed in the fuel checks variable. In the event-based PM variable of Matching, we removed 25/81 (31%) observations, all due to instruction recall failure. In the time-based variable of Matching 37/84 (44%) observations were removed, of which 35 due to instruction recall failure.

***Bayesian analyses***

BF_10_ indicates the likelihood of the observed data if the alternative hypothesis holds, in proportion to its likelihood if the null hypothesis is true. BF_10_ is mathematically defined as follows: BF_10_ = P(D|H1) / P(D|H0) where P is likelihood, D is data, and H1 and H0 are the alternative and null hypotheses, respectively. For example, if BF_10_ = 3, the data is three times more likely if the alternative hypothesis is true. Conversely, the inverted BF, i.e., BF_01_, indicates the likelihood of the data if the null hypothesis is true compared to if the alternative hypothesis is true. The BF_10_ is interpreted as follows: > 100 Extreme evidence for H1; 30 – 100 Very strong evidence for H 1; 10 – 30 Strong evidence for H1; 3 – 10 Moderate evidence for H1; 1 – 3 Anecdotal evidence for H1; 1 No evidence. The inverted BF is interpreted in the same way, but the evidence is for H0 (Jeffreys, 1961).

**Supplementary results**

***Blockwise performance***

*
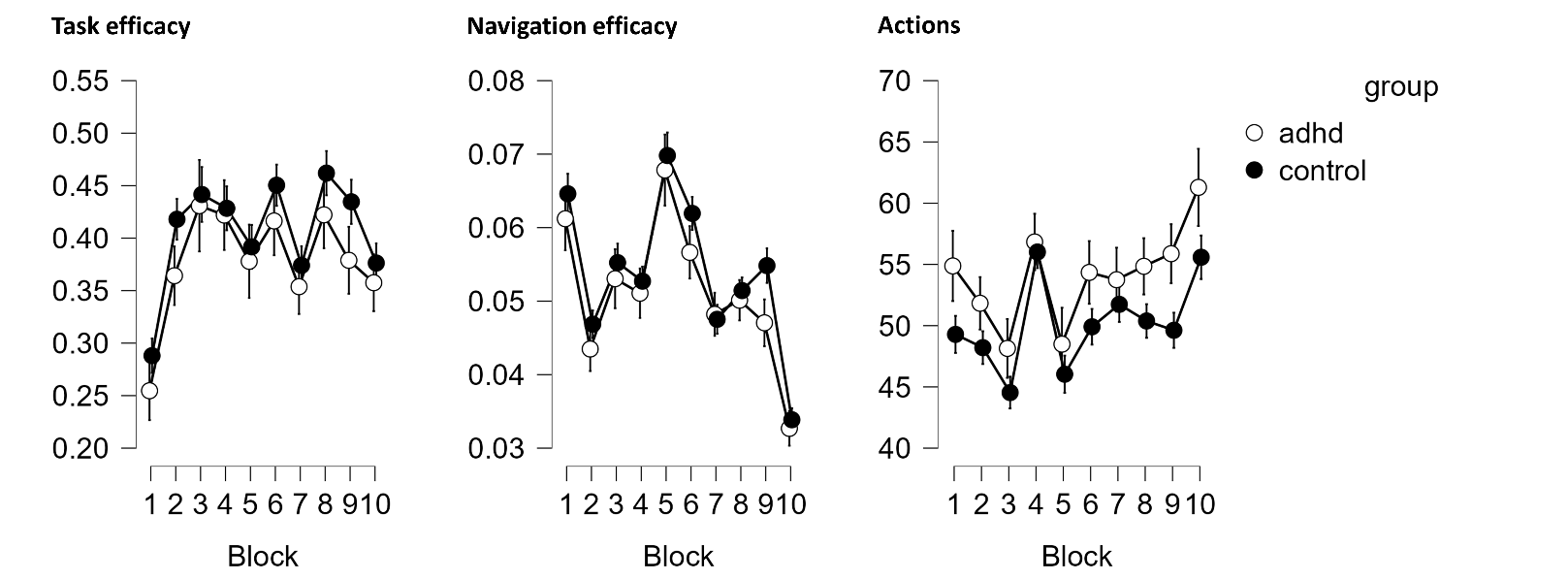
*

Figure S1. Blockwise performance in the planned EPELI variables that showed significant group differences in the main analysis. There was a main effect of Block in each variable, but no interaction between Group and Block.

***Ex-Gaussian analysis of intra-individual variability in response latencies***

The ex-Gaussian model assumes that a positively skewed response latency (RL) distribution can be represented as a mixture of exponential and gaussian distributions. The Gaussian component of the model is defined by parameters *mu* and *sigma*, which describe the mean and standard deviation of the normal distribution. The third parameter *tau* describes the mean of the exponential component (Leth-Steensen et al., 2000).

For EPELI data, the ex-Gaussian model was fit 3 times: for all RLs, for task-relevant RLs (where the second click was on an object relevant to the task), and for task-irrelevant RLs (where the second click was on an object irrelevant for completing the task). The ex-Gaussian model was fit for each participant’s RL distribution with R package “retimes” v. 0.1.2.

*Outliers exclusion for ex-Gaussian analysis*

The outlier exclusion was planned in two steps. First, in order to achieve a good fit of the ex-Gaussian model, abnormally long or short RTs were excluded for each participant separately based on the 1.5 interquartile range (IQR). Second, to avoid bias in statistical inference, participants with mean RT lying outside the 3 sigma interval were additionally excluded. The proportion of excluded data at the first step was 8.4%. The second step did not result in any exclusions.

After ex-Gaussian fit, the outliers with worst fit lying outside of 1.5 IQR in log-likelihood were excluded separately for each type of the clicks separately. The fit of the model measured by log likelihood did not differ between the groups for any type of the clicks after the exclusions.

| **Type of RL** | **Parameter** | **ADHD group** | **TD group** | **95% CI** | ***P*** |
| --- | --- | --- | --- | --- | --- |
| EPELI:  All clicks | *mu* | 0.85 | 0.95 | -0.18 to 0 | 0.048 |
|  | *sigma* | 0.37 | 0.41 | -0.08 to 0.01 | 0.091 |
|  | *tau* | 0.95 | 1.00 | -0.12 to 0.02 | 0.12 |
| EPELI:  Relevant  clicks | *mu* | 0.86 | 0.87 | -0.09 to 0.07 | 0.66 |
|  | *sigma* | 0.31 | 0.32 | -0.05 to 0.04 | 0.85 |
|  | *tau* | 0.86 | 0.91 | -0.12 to 0.02 | 0.13 |
| EPELI: Irrelevant  clicks | *mu* | 0.88 | 0.97 | -0.2 to 0 | 0.043 |
|  | *sigma* | 0.38 | 0.42 | -0.08 to 0.01 | 0.13 |
|  | *tau* | 0.96 | 1.00 | -0.11 to 0.03 | 0.25 |

Table S1. The results of ex-Gaussian modeling in the EPELI data.

The results of ex-Gaussian modelling of EPELI data are presented in the Table S1.

***References***

Adler, L. A., Spencer, T., Faraone, S. V., Kessler, R. C., Biederman, J., & Secnik, K. (2006). Validity of pilot Adult ADHD Self Report Scale (ASRS) to rate adult ADHD symptoms. *Annals of Clinical Psychiatry*, *18*(3), 145–148.

Ashton, M. C., Lee, K., & Goldberg, L. R. (2007). The IPIP-HEXACO scales: An alternative, public-domain measure of the personality constructs in the HEXACO model. *Personality and Individual Differences*, *42*(8), 1515–1526. https://doi.org/10.1016/j.paid.2006.10.027

Bravo, A. J., Villarosa-Hurlocker, M. C., Pearson, M. R., Prince, M. A., Madson, M. B., Henson, J. M., … McChargue, D. E. (2018). College student mental health: An evaluation of the DSM-5 Self-Rated Level 1 Cross-Cutting Symptom Measure. *Psychological Assessment*, *30*(10), 1382–1389. https://doi.org/10.1037/pas0000628

Conners, K. K., Epstein, J. N., Angold, A., & Klaric, J. (2003). Continuous performance test performance in a normative epidemiological sample. *Journal of Abnormal Child Psychology*, *31*(5), 555–562. https://doi.org/10.1023/A:1025457300409

Jeffreys, H. (1961). *Theory of Probability* (3rd ed.). Oxford: Oxford University Press.

Jylkkä, J., Ritakallio, L., Merzon, L., Kangas, S., Kliegel, M., Zuber, S., Hering, A., Salmi, J., & Laine, M. (2023). Assessment of goal-directed behavior with the 3D videogame EPELI. *PLOS ONE*, 18(3): e0280717.

Kerns, K. A. (2000). The CyberCruiser: An investigation of development of prospective memory in children. *Journal of the International Neuropsychological Society*, *6*(1), 62–70.

Leth-Steensen, C., Elbaz, Z. K., & Douglas, V. I. (2000). Mean response times, variability, and skew in the responding of ADHD children: a response time distributional approach. *Acta psychologica, 104(*2), 167–190. <https://doi.org/10.1016/s0001-6918(00)00019-6>

Rush, A. J., Trivedi, M. H., Ibrahim, H. M., Carmody, T. J., Arnow, B., Klein, D. N., … Keller, M. B. (2003). The 16-item Quick Inventory of Depressive Symptomatology (QIDS), clinician rating (QIDS-C), and self-report (QIDS-SR): A psychometric evaluation in patients with chronic major depression. *Biological Psychiatry*, *54*(5), 573–583. https://doi.org/10.1016/S0006-3223(02)01866-8

Voigt, B., Aberle, I., Schönfeld, J., & Kliegel, M. (2011). Time-based prospective memory in schoolchildren: The role of self-initiation and strategic time monitoring. *Zeitschrift Fur Psychologie / Journal of Psychology*, *219*(2), 92–99. https://doi.org/10.1027/2151-2604/a000053

Waris, O., Fellman, D., Jylkkä, J., & Laine, M. (2020). Stimulus novelty, task demands, and strategy use in episodic memory. *Quarterly Journal of Experimental Psychology*. https://doi.org/10.1177/1747021820980301
